# Bone marrow mesenchymal stem cell-derived CHI3L1 protected against *Helicobacter pylori*-induced gastric mucosal damage by promoting DNA damage repair *via* regulating CTCF/CPT1A

**DOI:** 10.1016/j.jbc.2026.113400

**Published:** 2026-08-07

**Authors:** Wang Qianqian, Wang Xiaoxiao, Wang Yongbin, Chen Jing, Hu Zhang

**Affiliations:** 1 Department of Laboratory Medicine, The Affiliated Hospital of Yangzhou University, Yangzhou University, Yangzhou, China; 2 School of Public Health, Yangzhou University, Yangzhou, China

**Keywords:** Helicobacter pylori, BMMSC-CM, gastric mucosal epithelial cells, DNA repair, fatty acid oxidation

## Abstract

*Helicobacter pylori* (*H*. *pylori*) infection triggers oxidative stress and persistent inflammatory responses, both of which lead to cellular DNA damage while impairing DNA repair. This constitutes a major factor in exacerbating inflammation and subsequently inducing precancerous lesions, with effective interventions still lacking. In our study, we found that bone marrow mesenchymal stem cell-conditioned medium (BMMSC-CM) could alleviate *H*. *pylori*-induced DNA damage in gastric mucosal epithelial cells and was further corroborated *in vivo* studies through immunofluorescence staining and flow cytometry. Screening *using solid*-*phase antibody arrays and transcriptome sequencing revealed that BMMSCs up*-*regulated CTCF expression* in gastric mucosal epithelial cells by secreting the glycoprotein CHI3L1, thereby enhancing Rad51 expression and promoting cellular DNA repair. Meanwhile, untargeted metabolomics and lipidomics further demonstrated that CTCF participated in the DNA repair process through CPT1A-mediated fatty acid oxidation. This study elucidated the role and application value of BMMSC-derived CHI3L1 in treating *H*. *pylori*-induced gastric mucosal damage. By exploring the specific mechanism through the regulation of fatty acid oxidation on DNA repair, this study provided new insights and potential therapeutic targets for treating *H*. *pylori* infection-related diseases.

*Helicobacter pylori* (*H*. *pylori*), as one of the most commonly transmitted Gram-negative bacteria in humans, affects approximately half of the world's population (1). However, the continuous increase in the resistance rate of antibiotics made it difficult to eradicate *H*. *pylori*, and there was also a lack of effective intervention measures for precancerous lesions such as gastric mucosal atrophy and even intestinal metaplasia after eradication (2). Therefore, the development of new therapies for *H*. *pylori*-induced gastric mucosal injury and the elucidation of underlying mechanisms were crucial for improving the treatment of *H*. *pylori*-related chronic gastritis and for devising effective strategies against gastric cancer precancerous lesions (3). The current understanding of *H*. *pylori* pathogenesis mainly focused on several key mechanisms. First, the bacteria promoted excessive production of reactive oxygen species (ROS) and reactive nitrogen species (RNS), leading to oxidative stress. Second, *H*. *pylori* induced DNA damage while impairing its repair mechanisms (4). Additionally, *H*. *pylori* triggered infiltration of inflammatory cells and overexpression of inflammatory factors, resulting in chronic inflammation (5). Furthermore, the pathogen interfered with normal cell cycle progression and disrupted pathways related to apoptosis and autophagy. *H*. *pylori* also influenced epigenetic modifications and altered the immune microenvironment (6). Importantly, the persistent inflammation and ongoing oxidative stress could cause metabolic disturbances in cells, which in turn further elevated ROS levels (7). Eventually, *H*. *pylori* caused various types of DNA damage, such as DNA oxidative damage, single-stranded or double-stranded DNA breaks, ribosome damage, and microsatellite instability (8). Consequently, the resulting genetic disturbances altered DNA repair pathways, leading to inaccurate DNA repair and genomic instability. This, in turn, promoted the development of gene mutations and chromosomal aberrations, thereby accelerating the malignant transformation of gastric epithelial cells (9). The evidence highlighted the pivotal role of unrepaired *H*. *pylori*-mediated DNA damage in fueling both inflammatory progression and cancer induction.

In recent years, mesenchymal stem cells (MSCs), based on their multidirectional differentiation potential and directional chemotaxis ability towards the damaged site, have been widely applied in the research of diseases of various systems, including inflammatory bowel disease, liver cirrhosis and myocardial ischemia after infarction (10). In cells infected with *H*. *pylori*, DNA damage was primarily repaired through pathways such as homologous recombination (HR), non-homologous end joining (NHEJ), mismatch repair, and base excision repair, with HR being the most accurate (11, 12). Recently, researchers have performed RNA sequencing on *H*. *pylori*-infected cells and found that HR repair-related genes such as NBS1, Rad51, and MRE11 were significantly down-regulated (7). A study on the combined effect of *H*. *pylori* infection and genetic variation published in The New England Journal of Medicine pointed out that among the 9 genes highly associated with the malignant transformation of gastric mucosa, 4 could mediate cellular HR repair (13). Han *et al.* demonstrated that *H*. *pylori* infection altered the cellular DNA repair balance, favoring error-prone NHEJ over high-fidelity HR, thereby promoting genomic instability (14). Collectively, these findings highlighted the critical role of the HR repair system in mitigating *H*. *pylori*-induced DNA damage and inflammation in gastric epithelial cells, offering a novel therapeutic avenue for treating *H*. *pylori*-related gastritis and preventing precancerous lesions. However, the specific component in BMMSC-CM and the precise mechanism through which they mediated the protective effect against *H*. *pylori*-induced gastric mucosal damage remained to be elucidated.

In this study, we aimed to investigate the therapeutic potential of BMMSC-CM against *H*. *pylori*-induced gastric mucosal damage. We have elucidated the molecular mechanisms by which BMMSC-CM enhanced DNA repair in gastric epithelial cells, thereby identifying novel targetable pathways to mitigate and reverse the damage.

## Results

### BMMSC-CM decreased DNA damage, protecting against *H*. *pylori*-induced gastric mucosal lesion

**Figure 1:**
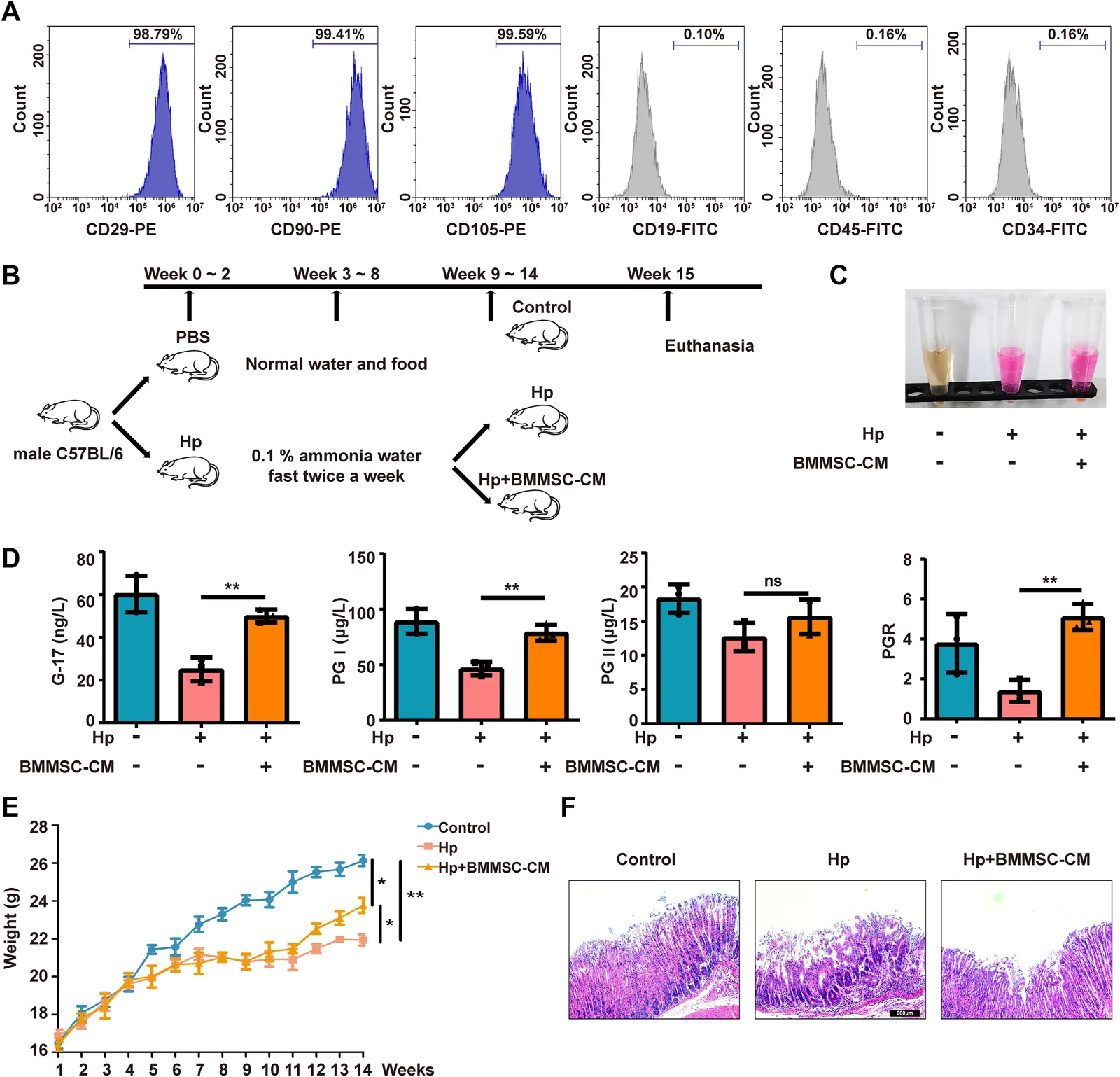
**BMMSC-CM protected against *H*. *pylori*-induced gastric mucosal lesion.***A*, characteristic surface markers of BMMSC were detected by flow cytometry analysis. *B*, schematic diagram of *in vivo* experiments. *C*, Rapid urease test. *D*, the expression levels of G-17, PG I, PG II and PGR in mouse serum. *E*, weight of mice. *F*, H&E staining of the stomach (scale bar: 200 μm). ∗*p* < 0.05, ∗∗*p* < 0.01.

To explore the impact of BMMSC on *H*. *pylori*-induced gastric mucosal lesion, male C57BL/6 mice were utilized to establish the *H*. *pylori* infection model for inducing gastric mucosal damage. BMMSC was isolated from healthy donors, phenotypically characterized by flow cytometry (Fig. 1*A*) and BMMSC-CM was delivered to the experimental mice by tail vein intravenous injection (Fig. 1*B*). Compared with the control group, the rapid urease test in the model group presented a positive reaction (Fig. 1*C*). To evaluate the extent of gastric mucosal injury in mice, we measured the serum levels of gastrin-17 (G-17), pepsinogen I (PG I), and pepsinogen II (PG II), which reflected the extent of gastric mucosal damage and atrophy. In contrast to the *H*. *pylori*-treated group, the BMMSC-CM group exhibited significantly higher G-17, PG I and PGR (PG I/PG II) levels (Fig. 1*D*). Since one of clinical manifestations of *H*. *pylori* infection-related gastric mucosal damage was the significant loss of body weight, we also measured the body weight of mice. As shown in Figure 1*E*, *H*. *pylori* infection slowed weight gain in mice, while the body weight of mice in the Hp + BMMSC-CM group was significantly higher than that in the *H*. *pylori* treatment group. Furthermore, a histopathological examination of the stomach was performed to directly demonstrate the therapeutic effects of BMMSC-CM against *H*. *pylori* infection-related gastric mucosal damage (Fig. 1*F*).

**Figure 2:**
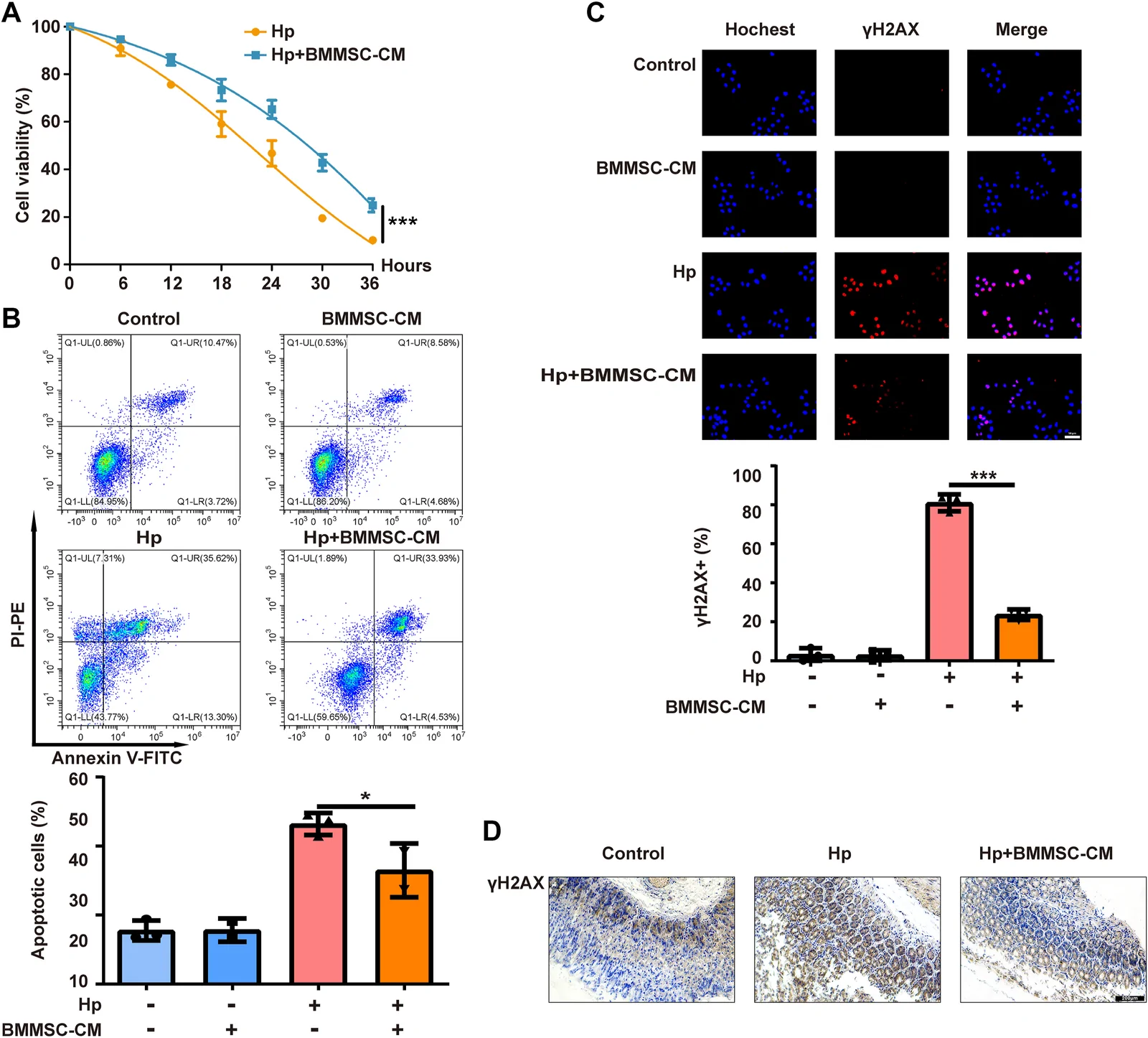
**BMMSC-CM decreased DNA damage induced by *H*. *pylori*.***A*, cell viability of GES-1 cells treated with *H*. *pylori* and BMMSC-CM were detected by CCK-8 assay. *B*, annexin V-FITC/PI flow cytometry assay was used to analyze the apoptosis rate of GES-1 cells. *C*, formation of γH2AX foci was detected by immunofluorescence (scale bar: 50 μm). *D*, IHC staining of γH2AX in tissues (scale bar: 200 μm). ∗*p* < 0.05, ∗∗∗*p* < 0.001.

After *in vitro* infection of GES-1 cells with *H*. *pylori* strain ATCC 43,504, BMMSC-CM was added for co-culture. The results of the CCK-8 assay and flow cytometry showed that BMMSC-CM led to a higher cell viability rate and a lower percentage of apoptotic cells after *H*. *pylori* treatment, suggesting that BMMSC-CM protected GES-1 cells from *H*. *pylori* infection (Fig. 2, *A* and *B*). To further investigate the impact of BMMSC-CM on DNA damage, we detected the expression of γH2AX in GES-1 cells and mouse gastric tissues. The proportion of γH2AX-positive cells, an indicator of double-strand breaks, was significantly higher than that in cells co-cultured with BMMSC-CM (Fig. 2*C*). Results of immunohistochemistry further demonstrated that BMMSC-CM could reduce *H*. *pylori*-induced DNA damage in gastric mucosal cells (Fig. 2*D*), protecting against *H*. *pylori*-induced gastric mucosal lesions. To further validate the effect of BMMSC-CM, we constructed a prokaryotic expression system for *CagA*, a major virulence factor of *H*. *pylori* that played a critical role in inducing gastric mucosal cell damage. GES-1 cells were transfected with pcDNA-*CagA* and pcDNA-NC. In line with the preceding data, the BMMSC-CM + pcDNA-CagA combination resulted in a markedly higher OD value and reduced cell apoptosis compared to the pcDNA-NC control. The BMMSC-CM + pcDNA-NC group did not differ significantly from the pcDNA-NC group alone (Fig. S1).

### BMMSC-CM promoted DNA repair through up-regulating CTCF in gastric mucosal epithelial cells in response to *H*. *pylori* infection

**Figure 3:**
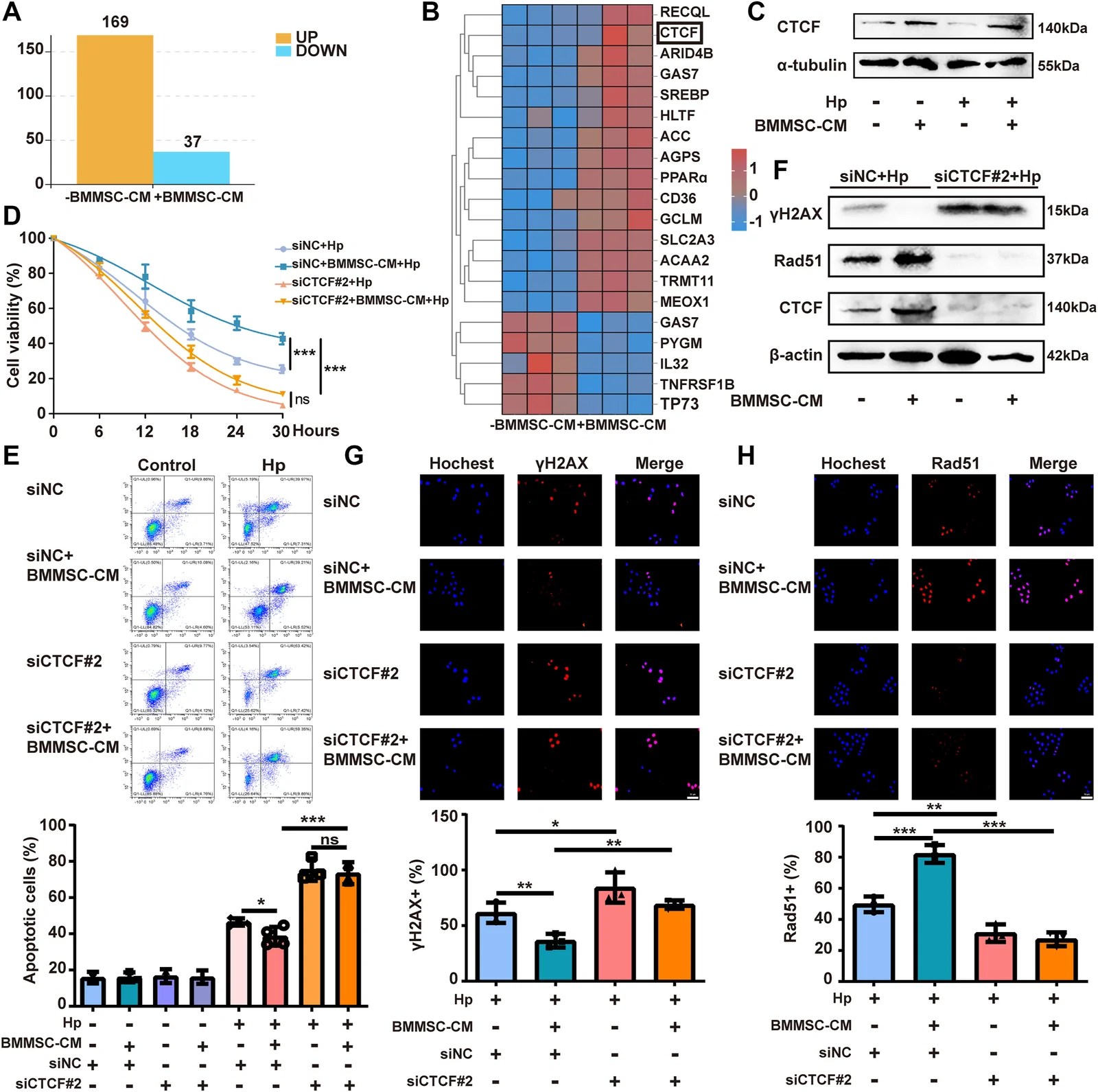
**BMMSC-CM promotes DNA repair through up-regulating CTCF in gastric mucosal epithelial cells in response to *H*. *pylori* infection.***A*, the RNA sequencing results showed that 169 genes were upregulated and 37 genes were down-regulated in the BMMSC-CM treatment group compared with the control group. *B*, Heatmap of different genes of GES-1 cells treated with or without BMMSC-CM. *C*, the expression of CTCF was detected by Western blot. *D*, Cell viability of GES-1 cells were detected by CCK-8 assay. *E*, Annexin V-FITC/PI flow cytometry assay was used to analyze the apoptosis rate of GES-1 cells. *F*, the expression of CTCF, Rad51 and γH2AX were detected by Western blot. *G* and *H*, formation of γH2AX and Rad51 foci were detected by immunofluorescence (scale bar: 50 μm). ∗*p* < 0.05, ∗∗*p* < 0.01, ∗∗∗*p* < 0.001.

We collected culture supernatants from GES-1 cells both before and after BMMSC-CM treatment and the RNA-seq analysis was performed to screen for potential BMMSC-CM-regulated genes in GES-1 cells. Compared with the control group, the BMMSC-CM treatment group exhibited 169 up-regulated and 37 down-regulated genes (Fig. 3*A*). Among these, DNA repair-related genes such as CTCF and RECQL were significantly up-regulated (Fig. 3*B*). Results of Western blot also showed that the expression of CTCF in GES-1 cells was obviously increased after BMMSC-CM treatment when exposed to *H*. *pylori* (Fig. 3*C*). Therefore, we reasonably speculated that BMMSC-CM may upregulate CTCF expression in GES-1 cells, thereby influencing the DNA damage repair process. Then, siRNAs targeting CTCF were transfected into GES-1 cells. Following *H*. *pylori* infection of GES-1 cells, the si-CTCF + BMMSC-CM group showed significantly reduced cell viability and increased apoptosis compared to the BMMSC-CM group (Fig. 3, *D* and *E*). In our preliminary studies, we found that CTCF was closely associated with DNA homologous recombination repair (HRR) and Rad51 was the pivotal repair factor in HRR (15). As shown in Figure 3*F*, the knockdown of CTCF showed significantly lower Rad51 expression levels, resulting in higher γH2AX levels. Immunofluorescence analysis yielded similar results (Fig. 3, *G* and *H*), confirming that CTCF was the critical mediator of DNA damage repair and essential for the protective efficacy of BMMSC-CM against *H*. *pylori*-mediated mucosal damage.

### CHI3L1 derived from BMMSC was necessary for promoting DNA repair of gastric mucosal epithelial cells

**Figure 4:**
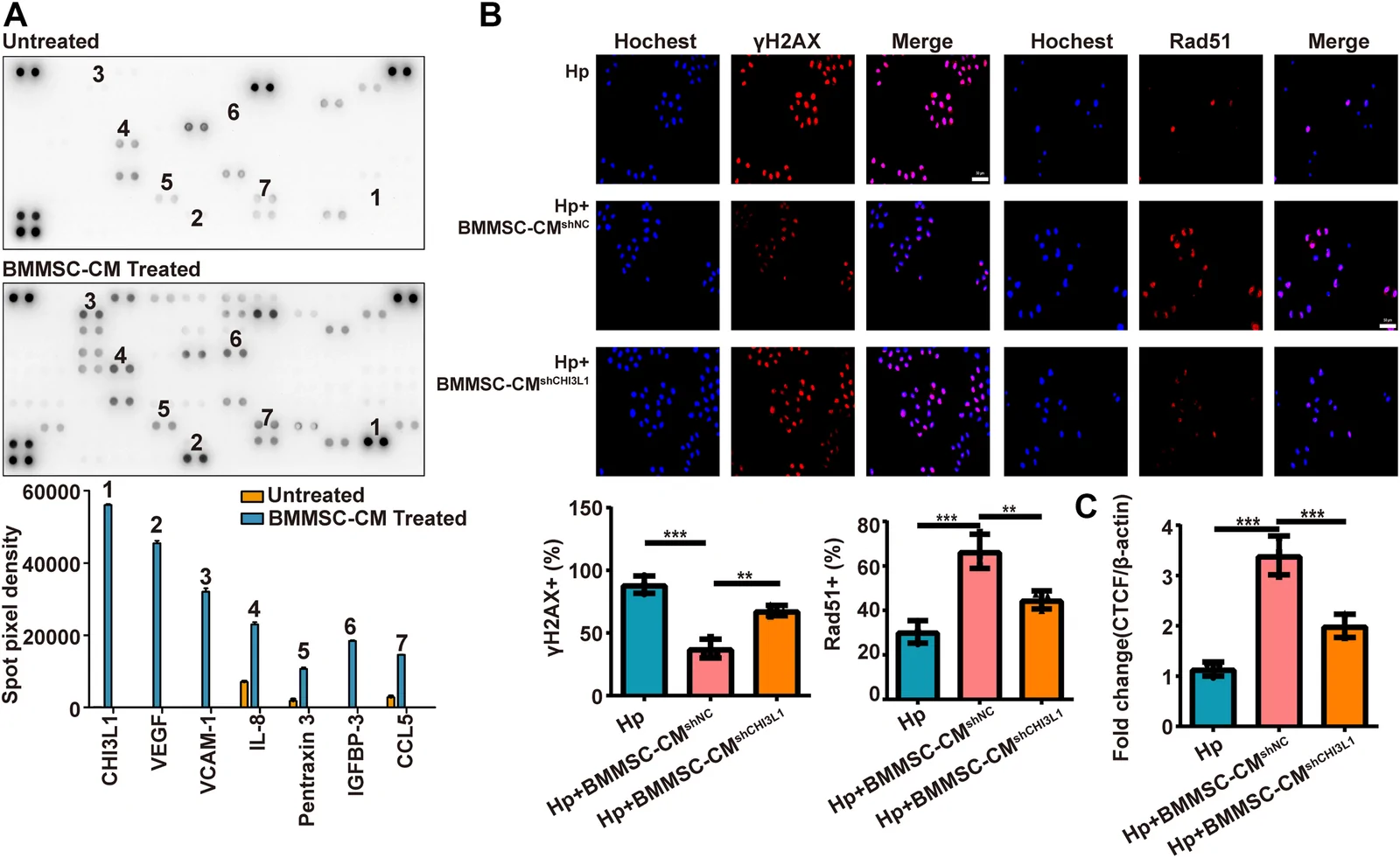
**CHI3L1 derived from BMMSC is necessary for promoting DNA repair of gastric mucosal epithelial cells.***A*, GES-1 cells were treated with or without BMMSC-CM and profiles of mean spot pixel density were detected. *B*, formation of γH2AX and Rad51 foci were detected by immunofluorescence (scale bar: 50 μm). *C*, the expression of CTCF were detected using qRT-PCR. ∗∗*p* < 0.01, ∗∗∗*p* < 0.001.

To identify the specific component of BMMSC-CM responsible for enhancing DNA repair in GES-1 cells, we screened the cell culture supernatants of both BMMSC and GES-1 cells for a range of soluble proteins, including cytokines, chemokines, and growth factors, using a Proteome Profiler Human XL Cytokine Array kit. Several cytokines showed significant differences between BMMSC and GES-1 cells, including CHI3L1, VEGF, VCAM-1 and IL-8 (Fig. 4*A*). Among these, CHI3L1 has been demonstrated to be closely associated with DNA damage repair (16). Consequently, BMMSC-derived CHI3L1 may represent a key mechanism in protecting cells from *H*. *pylori*-related genotoxic stress. Therefore, we used lentivirus to perform CHI3L1 knockdown in BMMSC, and the supernatant of BMMSC with CHI3L1 knockdown was collected and applied to GES-1 cells. The immunofluorescence analysis confirmed that BMMSC-derived CHI3L1 was essential for promoting DNA repair in GES-1 cells (Fig. 4*B*). In Figure 3, we have demonstrated that BMMSC-CM protects gastric mucosa from *H*. *pylori*-induced DNA damage by up-regulating CTCF. Here, we uncovered a crucial role for CHI3L1 in DNA damage repair in BMMSC-CM. Based on these observations, we proposed the hypothesis that the regulatory effect of BMMSC-CM on CTCF was associated with CHI3L1. qRT-PCR and Western blot assays were used to validate this hypothesis. Notably, when CHI3L1 in BMMSCs was knocked down, the increases in CTCF and Rad51 expression in GES-1 cells were both significantly abrogated (Figs. 4*C* and S2).

### CTCF contributed to DNA repair by regulating fatty acid oxidation through CPT1A

**Figure 5:**
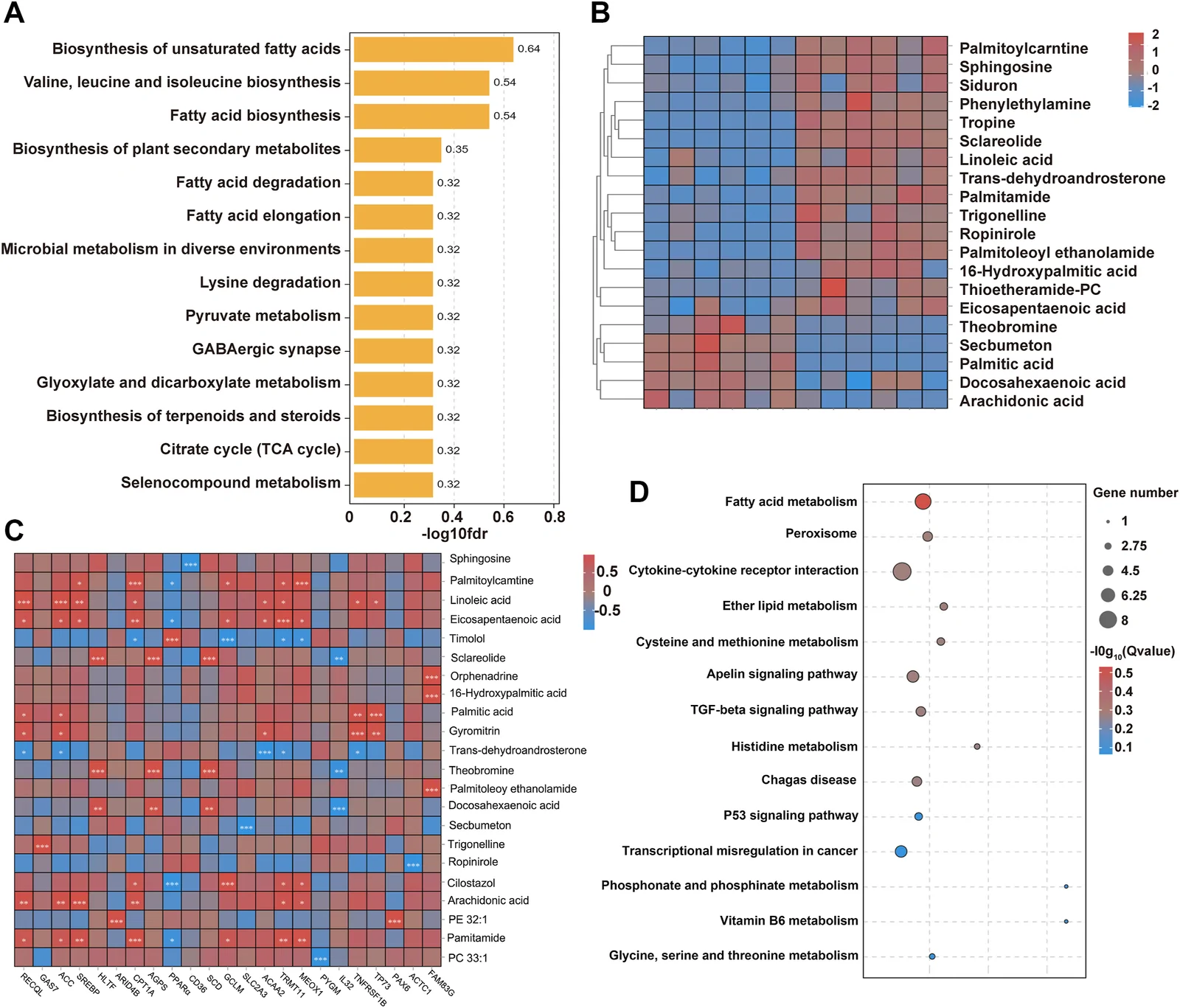
**Disturbances induced by BMMSC-CM treatment based on combined analysis of RNA-seq and non-targeted metabolomics.***A*, differential metabolite heatmap analysis. *B*, differential metabolite pathway enrichment analysis. *C* and *D*, combined transcriptomics and metabolomics analysis. *C*, correlation analysis. *D*, pathway integration analysis.

To further investigate the specific mechanism by which BMMSC-CM modulated CTCF to influence DNA repair, we first performed non-targeted metabolomics analysis on GES-1 cells before and after BMMSC-CM treatment. The OPLS-DA model was employed to elucidate variable differences between BMMSC-CM and the control group. The OPLS-DA score plot demonstrated the model's excellent fit (R^2^Y) and predictive capability (Q^2^) (Fig. S3, *A* and *B*). Response permutation tests (n = 200) confirmed that the model exhibited no overfitting (Fig. S3, *C* and *D*). Additionally, one-way ANOVA was applied to distinguish metabolites exhibiting the most pronounced differences. Based on metabolite screening criteria (VIP>1 and FDR-adjusted *p*-value <0.05), 20 metabolites with significant differences in cells were identified (Fig. 5*A*). Differential metabolite pathway enrichment analysis revealed that unsaturated fatty acid metabolism, valine, leucine, and isoleucine biosynthesis, fatty acid synthesis, and fatty acid degradation were the primary metabolic pathways (Fig. 5*B*). Further, the integrated analysis of RNA-seq and metabolomics data revealed that fatty acid metabolism disruption constituted the primary signaling pathway disturbance, with significantly correlated differences in fatty acid metabolites and associated regulatory genes (Fig. 5, *C* and *D*).

**Figure 6:**
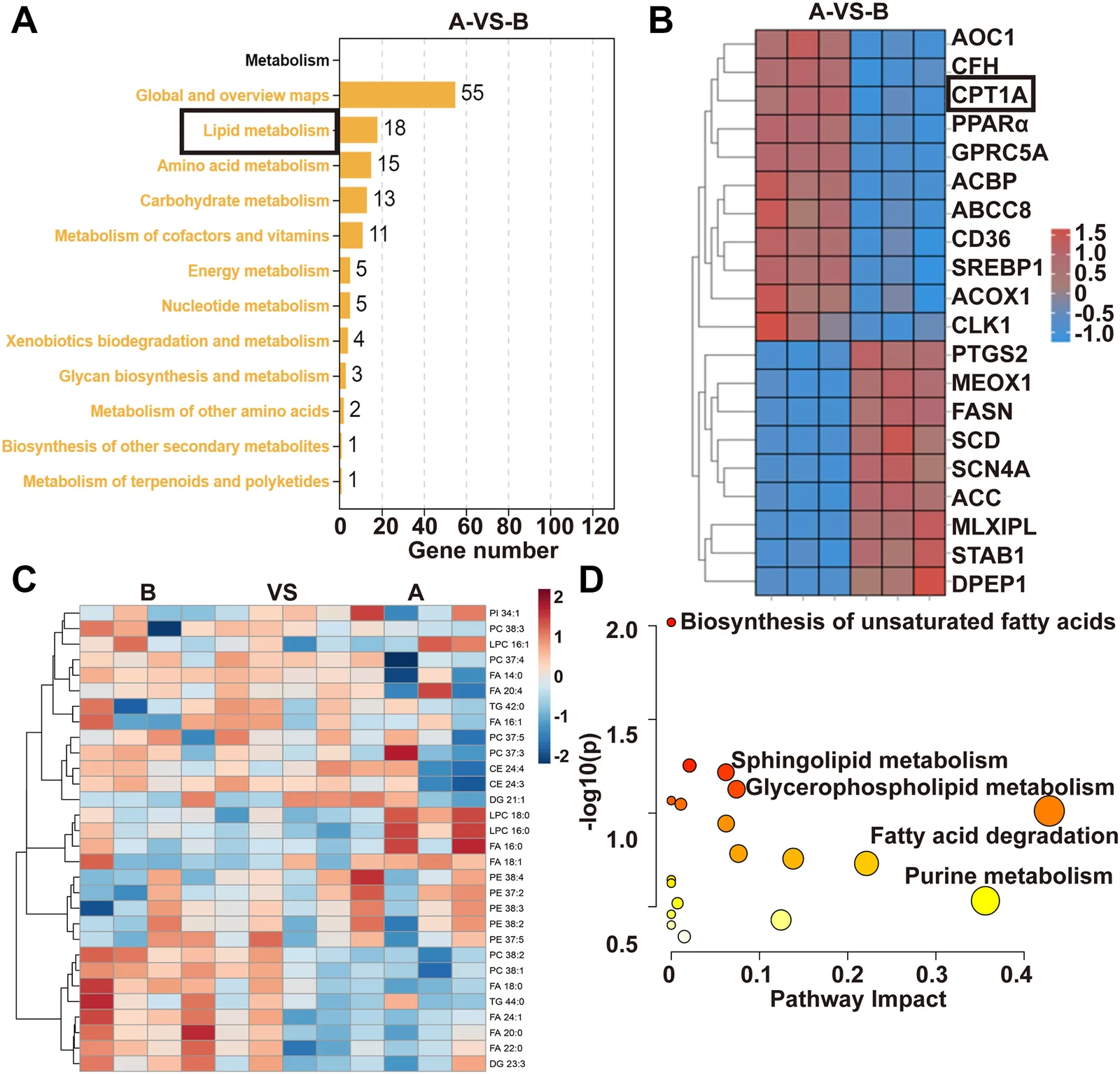
**CTCF was closely linked to lipid metabolism, especially fatty acid oxidation.***A*, GO enrichment analysis. *B*, differentially expressed gene heatmap analysis. *C* and *D*, differential metabolites and pathway enrichment analysis in lipidomics. A:shNC GES-1 cells,B:shCTCF GES-1 cells.

Based on this, we used lentivirus to perform CTCF knockdown in GES-1 cells and performed RNA-seq sequencing. GO analysis revealed that the major differentially expressed genes were closely associated with lipid metabolism disorders, particularly those related to fatty acid metabolism (PPARα, CD36, SERBP1, ACOX1, FASN, SCD). Notably, CPT1A, the rate-limiting enzyme for fatty acid oxidation (FAO), showed a significant down-regulated trend following CTCF knockdown (Fig. 6, *A* and *B*). Furthermore, we performed targeted lipidomics analysis on the cells after CTCF knockdown. The score plot results from the OPLS-DA model revealed distinct separation between CTCF low-expressing cells and the control cells, demonstrating excellent model fit and predictive performance (Fig. S4, *A* and *B*). The model validation was assessed using 200 permutations, confirming the model's predictive capability (Q2< 0, Fig. S4, *C* and *D*). The KEGG results demonstrated remarkable consistency with transcriptomic findings, revealing that differentially expressed lipid metabolites were enriched in fatty acid metabolism signaling pathways. In addition, the levels of most differential fatty acid metabolites exhibited downward trends, further indicating that CTCF was closely associated with the FAO process mediated by CPT1A (Fig. 6, *C* and *D*).

**Figure 7:**
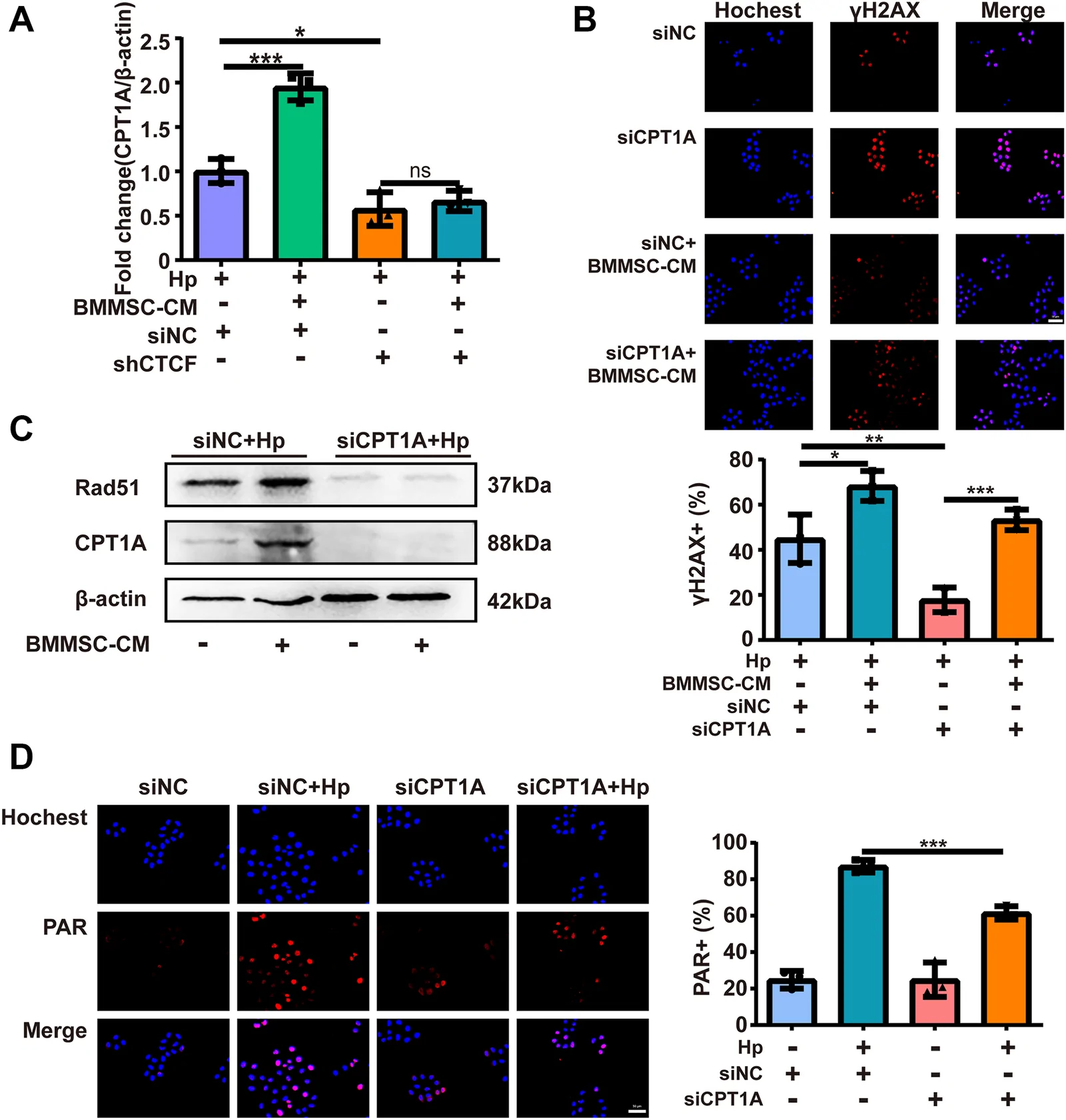
**CTCF contributed to DNA repair by regulating fatty acid oxidation through CPT1A.***A*, the expression of CPT1A were detected using qRT-PCR. *B*, formation of γH2AX and Rad51 foci were detected by immunofluorescence (scale bar: 50 μm). *C*, the expression of CPT1A and Rad51 were detected using Western blot. *D*, the expression of PAR was detected by immunofluorescence (scale bar: 50 μm). ∗*p* < 0.05, ∗∗*p* < 0.01, ∗∗∗*p* < 0.001.

Results of qRT-PCR confirmed that the expression of CPT1A increased under the influence of BMMSC-CM but significantly decreased in CTCF-depleted cells (Fig. 7*A*). We further verified the RNA-seq results using Western blot. Upon suppression of CTCF expression, CPT1A expression in GES-1 cell was also significantly decreased (Fig. S5). To further elucidate the role of CPT1A in BMMSC-CM promoting DNA damage repair caused by *H*. *pylori*, we suppressed CPT1A expression in GES-1 cells through siRNA transfection. The Western blot and immunofluorescence analysis showed that inhibiting CPT1A expression attenuated the promotive effect of BMMSC-CM on the DNA repair protein Rad51 and exacerbated the extent of DNA damage in GES-1 cells induced by *H*. *pylori* (Fig. 7, *B* and *C*). Taken together, these findings indicated that BMMSC-derived CHI3L1 enhanced DNA repair protein Rad51 *via* CTCF up-regulation in GES-1 cells, likely through CPT1A-dependent modulation of FAO pathway has recently been implicated in the DNA damage response, but its role in regulating DNA repair remains unresolved. In this study, we used etomoxir (ETO), which specifically impairs the import of fatty acids into mitochondria by inhibiting CPT1A, to further demonstrate FAO was required for BMMSC-CM promoting DNA repair. Notably, when cells were treated with ETO, the increase of Rad51 and CPT1A expression by BMMSC-CM was significantly abrogated. In addition, the repression of γH2AX expression by BMMSC-CM was also abrogated (Fig. S6). Next, we further investigated how CPT1A affects Rad51. Rad51 is a key molecule in homologous recombination (HR) repair. In the HR repair process, PARP1 is one of the first signaling proteins recruited to DNA breaks and facilitates the recruitment of DNA repair factors, such as BRCA1, by promoting PAR production. Moreover, it has been reported that FAO inhibition modulates PARP1 activity, as assessed by measuring PARylation levels under DNA-damaged conditions. In our study, we observed that the intensity of nuclear PAR was increased upon *H*. *pylori* treatment. Interestingly, this induction of PAR was substantially diminished in CPT1A-knockdown cells (Fig. 7*D*). Collectively, these results suggested that FAO-mediated PAR regulation upon DNA damage may represent a general phenomenon and further demonstrated that FAO appears to function as a significant metabolic regulator in the cellular response to DNA damage.

## Discussion

Recently, researchers have confirmed that MSC improved gastrointestinal tract injury through tail vein injection, oral administration, and submucosal injection (17). It was widely recognized that MSC mainly exerted the biological regulatory functions through paracrine effects. As a noncellular component, BMMSC-CM had the advantages of easy preservation, easy transportation, easier control of concentration and dosage, and higher safety (10). Therefore, we collected BMMSC-CM from healthy sources and co-cultured it with normal gastric mucosal epithelial cells. The results showed that when cells were treated with *H*. *pylori*, the cell viability of the BMMSC-CM group was facilitated compared with the control group. The DNA damage was significantly reduced and further verified *in vivo* experiments. At the same time, we used a solid-phase antibody microarray to detect and screen 105 cytokines in the culture supernatant of BMMSC-CM and gastric mucosal epithelial cells. Among the 7 factors with the most significant differences, CHI3L1 secreted by BMMSCs had the closest relationship with DNA damage repair. As a secreted glycoprotein, CHI3L1 was highly expressed in MSCs derived from multiple sources, including bone marrow and umbilical cord. It participated in key biological processes such as inflammatory response regulation, immunosuppression, angiogenesis, tissue repair, and remodeling (18). Given these multifunctional roles, CHI3L1 was implicated in a range of pathological conditions, including inflammatory diseases, neurodegenerative disorders, and various cancers (19). Current studies have found that umbilical cord-derived MSCs secrete a large amount of CHI3L1 to play a protective role in liver injury (20). Also, CHI3L1 regulates the DNA damage repair response and induces resistance to temozolomide in gliomas (21). However, the role of CHI3L1 derived from BMMSC in *H*. *pylori*-related diseases has not been clarified yet. The results of our research group showed that after knocking out the expression of CHI3L1 in BMMSC, the effect of its supernatant on the DNA damage repair of gastric mucosal cells was significantly weakened (22). Our results suggested that BMMSC could promote the DNA repair ability of gastric mucosal cells by secreting CHI3L1, thereby reducing *H*. *pylori*-related gastric mucosal damage.

In this study, we utilized high-throughput sequencing to analyze transcriptomic alterations in *H*. *pylori*-infected cells after treatment with BMMSC-CM. Among the genes exhibiting the most significant differential expression, CTCF stood out due to its strong association with the DNA damage repair response and showed a positive correlation with CHI3L1 expression in BMMSC-CM. CTCF was a zinc finger protein widely present in eukaryotes, and it acted as an insulator by changing the formation of chromatin loop domains (23). In addition, CTCF could enter the nucleus through the nuclear pore complex and played the primary role of multifunctional transcriptional regulators, thus participating in various processes such as the regulation of cell growth and differentiation (24). Recent studies revealed that ionizing radiation-induced DNA double-strand breaks were not randomly distributed but selectively enriched around CTCF-binding sites in a nuclease-dependent manner, and cells with low CTCF expression were more sensitive to DNA damage inducers (25). These research all indicated the close relationship between CTCF and DNA damage repair. However, the role of CTCF in *H*. *pylori*-related diseases has not been reported so far. Our study revealed that CTCF inhibition in gastric mucosal epithelial cells significantly reversed BMMSC-CM–mediated protection against *H*. *pylori*–induced gastric mucosal damage, thereby also compromising cellular DNA repair efficiency. In brief, the up-regulation of CTCF expression in gastric mucosal epithelial cells by CHI3L1 derived from BMMSC may be the vital key to promote DNA repair.

Recently, researchers have successively revealed the direct connection between changes in cellular metabolism and DNA damage repair (26). Related studies pointed out that the accumulation of metabolites in cells could affect the recruitment of HR repair-related factors to the damaged site during the repair process of double-strand breaks (27). Abnormal metabolic alteration could induce the lactylation modification of the DNA damage repair protein Mre11, thus promoting HR repair (26). Studies further indicated that DNA damage induced the upregulation of the pentose phosphate pathway, which promoted nucleotide synthesis to ultimately support DNA repair (28). Inhibiting glutamine senescence also exerted important functions in cell cycle arrest and DNA repair (29). In the transcriptome sequencing results, we found that the differentially expressed genes were highly enriched in cell metabolism-related pathways. Therefore, we conducted the non-targeted metabolomics analysis of gastric mucosal epithelial cells after treatment with BMMSC-CM, and the results showed that the differential metabolites were significantly enriched in the lipid metabolism pathway. Recently, Liu *et al.* analyzed the metabolites of gastric mucosal samples with different degrees of lesions and found that almost all the metabolites related to gastric cancer were lipids, revealing a close connection between abnormal lipid metabolism and gastric mucosal lesions (30). Furthermore, we conducted the combined analysis of the transcriptome and lipidomics of *H*. *pylori*–infected gastric mucosal epithelial cells after knocking out CTCF. The results revealed that the differentially expressed genes were significantly enriched in the pathway related to fatty acid degradation, and the key rate-limiting enzyme CPT1A in FAO was significantly down-regulated. This study demonstrated that inhibition of CPT1A reduced the expression of key HR repair pathway proteins and concomitantly abrogated the up-regulating effect of BMMSC-CM on their expression. Additionally, we revealed the important impact of FAO on DNA repair through analyzing PAR expression. Studies indicated that following DNA damage, FAO could activate the key DNA repair protein PARP1 through the provision of acetyl-CoA, thereby facilitating the DNA repair process (31), which was consistent with our findings. Consequently, FAO-mediated PAR regulation upon DNA damage may represent a general phenomenon and FAO represented a crucial component in the maintenance of genomic stability. On the basis of our results, it will be important for future investigations to explore how FAO coordinately regulates both cell survival and genomic integrity upon genotoxic challenge. Further mechanistic exploration of the role of BMMSC-CM in DNA damage repair will also be pursued in our future studies.

In summary, our data indicated that when DNA double-strand breaks occurred in gastric mucosal epithelial cells due to *H*. *pylori* infection, the glycoprotein CHI3L1 secreted by BMMSC up-regulated the expression of CTCF in gastric mucosal epithelial cells. CTCF participated in the regulation of FAO through CPT1A. FAO could activate the key DNA repair protein PARP1 through the provision of acetyl-CoA, ultimately promoting DNA repair, reducing gastric mucosal damage and maintaining genomic stability. Our findings provided new targets and experimental evidence for the treatment of *H*. *pylori* infection-related diseases. By underpinning genomic stability and revealing avenues to prevent/treat precancerous lesions, our study offered a basis for important clinical applications.

## Experimental procedures

### Human BMMSC isolation

Human bone marrow specimens were obtained during surgery for orthopedic trauma patients in Affiliated Hospital of Yangzhou University. All procedures were approved by the Ethics Committee of Yangzhou University with informed consent from each patient. An equal volume of 1.077 g/ml Ficoll was added to bone marrow diluted with PBS and then centrifuged at 800*g* for 20 min to isolate primary BMMSC.

### Cell culture and preparation of BMMSC conditioned medium (BMMSC-CM)

BMMSC was cultured in Dulbecco’s Modified Eagle’s Medium (DMEM, Gibco, USA) containing 10% fetal bovine serum (FBS, Gibco) at 37 °C with 5% CO_2_. The normal gastric mucosal epithelial cells (GES-1) were purchased from the Chinese Academy of Sciences Type Culture Collection Committee Cell Bank (Shanghai, China) and cultured in RPMI-1640 (Biological Industries) with 10% FBS. When BMMSCs reached 70%∼80% confluence, the culture medium was replaced with fresh DMEM medium and further incubated for 48 h. The cell culture supernatant was collected and mixed with an equal volume of RPMI 1640 to obtain BMMSC-CM.

### *H*. *pylori* culture

The isolated *H*. *pylori* strain ATCC 43,504 was kindly provided by Prof. Yanbing Ding (Affiliated Hospital of Yangzhou University). The bacteria were maintained and grown on Columbia agar supplemented with 7% defibrinated sheep blood and incubated under microaerophilic conditions (5% O_2_, 10% CO_2_, 85% N_2_) at 37 °C. After 3 days of culturing, the *H*.*pylori* strain was collected for subsequent experiments.

### Construction of *CagA* eukaryotic expression plasmid

For the assay, the pcDNA3.1 (+) eukaryotic expression plasmid was obtained from Jie Rui Biological Company in Shanghai, China. The initial step involved digesting both the empty plasmid vector and the full-length CagA gene with EcoRI-XhoI. Following digestion, the vector and gene fragments were purified. A 20 μl ligation reaction was then assembled using the following components: 4 μl of the full-length CagA gene (digested with EcoRI-XhoI), 2 μl of the digested pcDNA3.1 vector, 1 μl of T4 DNA ligase, 2 μl of 10 × T4 buffer, and 11 μl of water. This mixture was incubated at 22 °C for 30 min to generate the initial CagA eukaryotic expression plasmid construct. Subsequently, the ligation product was transformed into XL10-Gold competent cells. Positive clones were identified through colony screening and verified by Sanger sequencing (Supplementary Files 1 and 2). Once successful construction was confirmed, the plasmid was purified for downstream applications using the GK2004 plasmid miniprep kit and the GoldHi EndoFree Plasmid Midi Kit (CW2581S).

### Cell transfection

siRNAs targeting CTCF (siCTCF#1: 5′-GUGCAAUUGAGAACAUUAUTT-3′; siCTCF#2: 5′-GTAGAAGTCAGCAAATTAA-3′) were transfected to cells using Lipofectamine 2000. shRNA was obtained from GenePharma Technologies (Shanghai, China). CTCF shRNA sequences (GCGAAGAATGACCACAAAT) were separately cloned into the pLKO.1 lentiviral vector (#10878, Addgene). shRNAs targeting CHI3L1(Forward:5′-CCGGATCAAGTCAGTACCGCCATTTCTCGAGAAATGGCGGTACTGACTTGATTTTTTG-3′,Reverse:5′-AATTCAAAAAATCAAGTCAGTACCGCCATTTCTCGAGAAATGGCGGTACTGACTTGAT-3′) were designed, synthesised (Sangon Biotech, Shanghai, China), and cloned into pLKO.1_TRC Cloning Vector. A non-targeting sequence construct was used as the negative control. Cells were infected with the virus and selected through 2 μg/ml puromycin (Beyotime). siRNAs for CPT1A (sense: 5′-CAUCCAUGCAUACCAAAGUTT-3′,antisense:5′-ACUUUGGUAUGCAUGGAUGTT-3′) and negative control (sense: 5′-UUCUCCGAACGUGUCACGUTT-3′,antisense:5′-ACGUGACACGUUCGGAGAATT-3′) were designed and constructed by GenePharma Technologies (Shanghai, China).

### Cell counting kit-8 (CCK-8) assay

Cells were infected with *H*. *pylori* (MOI = 100∶1) for 24 h and then co-cultured with BMMSC-CM. Cell viability was analyzed at 0, 6, 12, 18, 24, 30, and 36 h. Following transfection with pcDNA-CagA, cells were co-cultured with BMMSC-CM, and cell viability was assessed at 0, 6, 12, 18, 24, 30, and 36 h. Cell viability was analyzed by the Cell counting kit-8 assay (Vazyme) according to the manufacturer's instructions. Absorbance was measured at 450 nm using a microplate reader with cell-free wells serving as blank controls. Each CCK-8 assay was repeated three times.

### Flow cytometric apoptosis analysis

The cells were digested with EDTA-free trypsin and centrifuged at 300*g*, 4 °C for 5 min. After washing twice with PBS, 200 μl of 1× Binding Buffer, 3 μl of Annexin V-FITC, and 3 μl of PI staining solution (BD Biosciences) were added to each tube and mixed well. The samples were incubated at room temperature in the dark for 10 min. Stained samples were analyzed by flow cytometry (FACSCalibur) within 1 h, with an excitation wavelength of 488 nm. Annexin V-FITC green fluorescence was detected in the FL1 channel, and PI-PE red fluorescence was detected in the FL3 channel. The apoptosis rate was analyzed using FlowJo 7.6 software. Each flow cytometric apoptosis analysis was repeated three times.

### Western blot analysis

The total protein extraction procedure was performed according to the manufacturer's instructions of total protein extraction kit (CWBIO). Cells or tissues protein lysates in PIPA buffer (Invitrogen) were separated *via* 12% SDS-PAGE. After electrophoresis at 60 V for 2 h, the proteins were transferred from the SDS-PAGE gel to a PVDF membrane (350 mA, 2 h). The membrane was blocked with 50 g/L skim milk at room temperature for 2 h. Antibodies (diluted to 1:1000, Cell Signaling Technology, USA) were added and incubated overnight at 4 °C. The PVDF membrane was washed with TBST for 30 min, followed by incubation with goat anti-mouse or anti-rabbit secondary antibodies (diluted to 1:3000, Cell Signaling Technology) at 37 °C for 1 h. After washing with TBST, the signals were detected through ECL reagents (Millipore). Antibody: α-tubulin (Cell Signaling Technology), β-actin (Cell Signaling Technology), γH2AX (Cell Signaling Technology), CTCF (Cell Signaling Technology), Rad51 (Abcam), CPT1A (Abcam), PAR (Abcam).

### *In vivo* experiment

5∼6 weeks old 50 male C57BL/6 mice (Cavens) were raised in accordance with institutional guidelines of the animal facility at Yangzhou University. All experiments were performed according to the National Institutes of Health guide for the care and use of Laboratory animals (NIH Publications No. 8023, revised 1978) with the permission of the local ethics committee of Yangzhou University. Mice were randomly divided into the control group and model group. The model group was further subdivided into an *H*. *pylori* infection group and the *H*. *pylori* infection + BMMSC-CM treatment group, with 10 mice in the control group and 20 mice in each of the other two groups. After one week of acclimatization feeding, the *H*. *pylori* bacterial suspension was adjusted to a concentration of 1 × 10^9^ CFU/ml using PBS. Mice in the model group were pretreated with NaHCO_3_. On Tuesdays, Thursdays, and Saturdays each week, model group mice were orally gavaged with the *H*. *pylori* suspension (500 μl per mouse). Mice were fasted for 12 h before each gavage and for 4 h afterward. The control group received sterile PBS instead, for a total of six gavages. Concurrently, model group mice were provided with freshly prepared 0.1% ammonia water ad libitum as the drinking water. Eight weeks after the last gavage, one mouse from each group was randomly euthanized, and a rapid urease test was performed on gastric tissue to confirm *H*. *pylori* infection. Following confirmation, mice in the *H*. *pylori* infection + BMMSC-CM treatment group received tail vein injections of mouse-derived BMMSC-CM (200 μl per mouse) every other day. The control and *H*. *pylori* infection groups received sterile α-MEM medium instead. Body weight was recorded weekly during this period. Five weeks after initiating the BMMSC-CM injections, all mice were anesthetized, and serum samples were collected. Following euthanasia, the entire stomach was dissected from each mouse.

### Profiling proteins in cell culture supernates

The Human XL Cytokine Array (R&D Systems) detected multiple cytokines, chemokines, growth factors and other soluble proteins in cell culture supernates of GES-1 cells and BMMSC. 500 μl of cell culture supernate was run on each array. Profiles of mean spot pixel density were created using a transmission-mode scanner and image analysis software.

### H&E staining and Immunohistochemistry

Tissues from mice were formalin-fixed, paraffin-embedded, sectioned and stained with hematoxylin and eosin (H&E). For IHC staining, sections were stained with anti-γH2AX (diluted to 1∶100, Cell Signaling Technology) overnight at 4 °C and imaged with an AxioLab microscope equipped with an AxioCam HRC CCD camera (Zeiss).

### Immunofluorescence and image analysis

Cells were fixed with 4% paraformaldehyde for 20 min at room temperature, followed by three PBS washes (5 min each). Permeabilization was performed using 0.2% Triton X-100. Nonspecific binding was blocked with 5% bovine serum albumin (BSA) for 2 h at room temperature. Subsequently, cells were incubated overnight at 4 °C with 200 μl of primary antibodies against γH2AX (diluted to 1:200, Cell Signaling Technology), Rad51 and PAR (diluted to 1:100, Abcam). Anti-rabbit IgG Fab2 Alexa Fluor(R) 555 molecular probes at 37 °C for 1 h (Cell Signaling Technology). Nuclear counterstaining was completed with Hoechst 33342 (1 μg/ml, 10 min) prior to imaging. Glasses were imaged using a fluorescence microscope (Zeiss, Germany). Each specimen was counted blindly by 2 ∼ 3 laboratory technicians, and the mean and standard deviation were calculated. At least 40 cells were counted per specimen, and cells with exceeding 10 γH2AX or Rad51 foci were recognized as positive cells, and the positive cells per treatment group were counted for quantification. Each immunofluorescence analysis was repeated three times.

### RNA extraction and quantitative real-time reverse transcription-polymerase chain reaction(qRT-PCR)

Total RNA of GES-1 cells were extracted using TRIzol reagent (Invitrogen). cDNA synthesis and qRT-PCR were performed according to the instructions of HiFiScript cDNA Synthesis kit and the UltraSYBR Mixture (CWBIO). Primer sequences were: CAGTGGAGAATTGGTTCGGCA and CTGGCGTAATCGCACATGGA for CTCF; AAGCAACGATCACATCGACAC and TCAGGTTGGGGTTCCTGTTCT for CHI3L1; TCCAGTTGGCTTATCGTGGTG and TCCAGAGTCCGATTGATTTTTGC for CPT1A; GCAAAGACCTGTACGCCAACA and TGCATCCTGTCGGCAATG for β-actin. Each qRT-PCR analysis was repeated three times.

### RNA-seq analysis

The total RNA was extracted and delivered to the Genedenovo Biotechnology Company (Guangzhou, China) for transcriptome sequencing. In detail, cells were collected for cellular RNA extraction, with RNA purity and concentration measured. RNA quality, integrity, and absence of DNA contamination were assessed using an Agilent 2100 Bioanalyzer and agarose gel electrophoresis. Following RNA quality validation, the size of ligation products was screened *via* agarose gel electrophoresis. Finally, DNA/RNA/small RNA/cDNA libraries were sequenced using the Illumina HiSeq 6000 platform. Data quality control analysis was performed, low-quality reads were filtered to obtain clean reads, followed by base quality analysis, sequence alignment analysis, and gene analysis. Expression quantification, sample relationship analysis, and inter-sample differential analysis were conducted. Based on differential analysis results, genes meeting FDR <0.05 and |log2FC| >1 were identified as differentially expressed genes. All differentially expressed genes were mapped to the Gene Ontology (GO) database and KEGG public database. Pathway significance enrichment analysis was employed to determine the biological processes and signaling pathways involved in these differentially expressed genes.

### Non-targeted metabolomics analysis

Cells were collected for metabolite extraction. The cells in the culture dishes were washed three times with pre-cooled PBS, rapidly quenched with liquid nitrogen, and then scraped off with 500 μl of methanol/deionized water (4:1, v/v) and transferred into tubes. Each tube was subjected to bead milling for 3 min (50 Hz) with steel beads. After thawing with 200 μl of methanol/deionized water (4:1, v/v), the samples were centrifuged at high speed (12,000 rpm) for 15 min. The supernatant was collected for injection analysis. The mass spectrometry conditions and metabolite analysis were provided in Table S1.

### Cell lipidomics analysis

Lipidomics analysis was performed using an AB SCIEX Triple Quad 4500 LC-MS/MS system (AB SCIEX). Chromatographic separation was achieved using an ACQUITY UPLC BEH C18 column (2.1 × 100 mm, 1.7 μm; Phenomenex). After cell incubation, cells were collected for lipid metabolite extraction. The cells were washed in the culture dish three times with pre-chilled PBS, then rapidly quenched with liquid nitrogen. 100 μl serum was combined with 400 μl methanol/deionized water (3:1, v/v). Then, 1.0 ml of methyl tert-butyl ether (MTBE) was added, and the mixture was vortexed vigorously. After the addition of 500 μl deionized water to induce phase separation, the sample was placed on the ice bath for 15 min. The supernatant was subsequently centrifuged at 14,000*g* for 15 min at 4 °C to isolate the lipid layer. Finally, the dried extract was reconstituted in 200 μl of ACN/IPA/H_2_O (65:30:5, v/v/v) for mass spectrometric analysis. The mass spectrometry conditions and lipid metabolite analysis conditions (including internal standard information) were listed in Table S2.

### Statistical analysis

Data were expressed as the mean ± SD and analyzed by GraphPad Prism 7.0 software. Differences between two independent groups were analyzed with Student’s *t* test. One-way ANOVA combined with Newman-Keuls test were used to compare difference between more than two groups. *p* < 0.05 was considered to be statistically significant.

## Consent for publication

All authors agreed to publish the ultimate version of manuscript.

## Data availability

All data supporting the findings of this study were included within the paper and its supplementary information, and additional raw data were available from the corresponding author on request.

## Supporting information

This article contained the supporting information.

## Conflict of interest

The authors declare that they have no conflicts of interest with the contents of this article.
