# Supplementary material for: Bone marrow mesenchymal stem cell-derived CHI3L1 protected against Helicobacter pylori-induced gastric mucosal damage by promoting DNA damage repair via regulating CTCF/CPT1A
Source: J Biol Chem. 2026 Aug 7;302(9):113400. doi: 10.1016/j.jbc.2026.113400 (PMC13545433; doi:10.1016/j.jbc.2026.113400)
Supplement: Supplementary file 1 [file mmc1.docx]

**Supplementary Table S1. Parameters for metabolomics analysis**

| Mass Spectrometry System | Metabolite profiling was performed using a SCIEX Triple TOF 5600 mass spectrometer with a Waters ACQUITY UPLC BEH C18 column (1.7 μm, 2.1 mm × 50 mm), employing a 5 μL injection volume and a mobile phase flow rate of 0.3 mL/min. UPLC-MS/MS analysis employed ESI positive and negative ion modes for detection. Mobile phase conditions comprised A (100% ACN) and B (containing 0.1% formic acid for positive ion mode or 5 mM ammonium formate for negative ion mode). The gradient conditions were: 0–2 min, A:98%; 2–14 min, A:98%–2%; 14–17 min, A: 2%; 17–17.1 min, A: 2%–98%; 17.1–20 min, A: 98%. |
| --- | --- |
| Mass spectrometry parameters | The ion source voltage floating (ISVF) for positive and negative ions was 5 kV and -4.5 kV, respectively. The collision energy (CE) was ±35 eV, and the dissociation potential (DP) was ±80 eV. The heater temperature and curtain gas were 500°C and 35 psi, respectively. Ion source gases 1 and 2 were set at 50 psi. Column temperature was maintained at 40°C. The full scan m/z range is 40–1000. For quality control (QC), 5 μL of each sample was pooled. The QC sample sequence was loaded every 5 injections. |
| Metabolomics analysis | Raw LC-MS/MS data were processed online using XCMS Online. Following retention time correction and peak extraction, peak identification and metabolite characterization were applied for further validation. Features with missing values exceeding 20% or peak intensity RSD exceeding 30% were excluded. Data were normalized using metabolite-consistent internal standards, then analyzed with SIMCA-P software to perform principal component analysis (PCA), orthogonal partial least squares discriminant analysis (OPLS-DA), and 200 paired prediction analyses to evaluate metabolic analysis models. Features with VIP scores > 1.0 and intergroup P-values < 0.05 were identified as differential metabolites. Structural matching and validation were performed using the HMDB database based on m/z values and MS2 spectra. MetaboAnalyst 5.0 was employed to enrich metabolic pathways for differential metabolites using the KEGG pathway database. |

**Supplementary Table S2. Parameters for lipidomics analysis**

| Mass Spectrometry System | Lipidomics analysis was performed using a SCIEX Triple TOF 5600 mass spectrometry system and a Waters ACQUITY UPLC BEH C18 column (1.7 μm, 2.1 mm × 50 mm) at a column oven temperature of 40 °C. |
| --- | --- |
| Mobile phase | Mobile phases A and B were respectively ACN/H2O (6:4, v/v, containing 10 mM ammonium formate, 0.1% formic acid) and IPA/ACN (9:1, v/v, containing 10 mM ammonium formate, 0.1% formic acid). The flow rate was kept constant at 0.3 mL/min, and the injection volume was 5 μL. The linear gradient conditions were as follows: 0-2 min, A: 85%-70%; 2-5 min, A: 70%-40%; 5-14 min, A: 40%-20%; 14-16 min, A :20%-10%; 16-18 min, A: 10%; 18-18.1 min, A: 10%-85%; 18.1-20 min, A: 85%. |
| Mass spectrometry parameters | The ionization sources of positive and negative ion spray voltage floating (ISVF) were 5 kV and -4.5 kV respectively. The collision energy was ±35 eV and the declustering voltage was ±80 eV. The heating temperature and curtain gas were 500 °C and 35 psi respectively. Ion source gas1 and gas2 were 50 psi. Data acquisition was performed in full scan mode with an m/z range of 100-1000. |
| Lipid data analysis | LC-MS/MS raw data were imported into LipidView™ (SCIEX, USA) software for lipid identification. [M+H]+ and [M-H]- ions were extracted and identified in positive and negative ionization modes respectively. Metabolite identification bias range was 5.0 ppm. Lipid peak areas were normalized by IS (internal standard, 15:0–18:1(d7) PC, 15:0–18:1(d7) PE, 15:0–18:1(d7) PS, 15:0–18:1(d7) PG, 15:0–18:1(d7) PI, 15:0–18:1(d7) PA, 18:1(d7) LPC, 18:1(d7) LPE, 18:1(d7) Chol Ester, 18:1(d7) MG, 15: 0–18:1(d7) DG, 15:0–18:1(d7)-15:0 TG, 18:1(d9) SM.) and quantified by comparison with identified lipids. Finally, differential lipid biomarkers were screened by comparing ions to Lipidmaps and HMDB databases based on m/z mass values and MS2 spectra. |

**Supplementary Figures**

Fig. S1 (A) GES-1 cells were transfected with pcDNA-*CagA* and pcDNA-NC. Cell viability of GES-1 cells were detected by CCK-8 assay. (B) Annexin V-FITC/PI flow cytometry assay was used to analyze the apoptosis rate of GES-1 cells. ***P* < 0.01, ****P* < 0.001.

Fig. S2. Cells were exposed to Hp and treated with BMMSC-CM^shNC^ or BMMSC-CM^shCHI3L1^. The expression of CTCF and Rad51 were detected by western blot.

Fig. S3 (A-B) The OPLS-DA score plot demonstrated the model's excellent fit (R²Y) and predictive capability (Q²). (C-D) Response permutation tests (n = 200) confirmed the model exhibited no over fitting.

Fig. S4 The score plot results from the OPLS-DA model revealed distinct separation between CTCF low-expressing cells and the control cells. (A-B). The OPLS-DA score plots in positive mode (R²Y = 0.976, Q² = 0.46); negative mode (R²Y = 0.899, Q² = 0.49). (C-D). Permutation test results (n = 200) of positive mode (R²Y = 0.897, Q² = –0.04) and negative mode (R²Y = 0.826, Q² = –0.08).

Fig. S5. The expression of CTCF and CPT1A in GES-1 cells which were transfected with siNC or siCTCF were detected by western blot.

Fig. S6. Cells were exposed to Hp and treated with BMMSC-CM and etomoxir. The expression of CPT1A, γH2AX and Rad51 were detected by western blot.
